# The effects of removing dead bacteria by propidium monoazide on the profile of salivary microbiome

**DOI:** 10.1186/s12903-021-01832-5

**Published:** 2021-09-22

**Authors:** Qidi Ren, Fangqiao Wei, Chao Yuan, Ce Zhu, Qian Zhang, Junkang Quan, Xiangyu Sun, Shuguo Zheng

**Affiliations:** 1 Department of Preventive Dentistry, Peking University School and Hospital of Stomatology & National Center of Stomatology & National Clinical Research Center for Oral Diseases & National Engineering Laboratory for Digital and Material Technology of Stomatology, Beijing, People’s Republic of China; 2 Department of Preventive Dentistry, Shanghai Jiao Tong University School of Dentistry, Shanghai Ninth People’s Hospital, Shanghai, People’s Republic of China; 3 Central Laboratory, Peking University School and Hospital of Stomatology & National Center of Stomatology & National Clinical Research Center for Oral Diseases & National Engineering Laboratory for Digital and Material Technology of Stomatology, Beijing, People’s Republic of China

**Keywords:** Salivary microbiome, Propidium monoazide, Removal of dead bacteria, *16S rRNA* sequencing

## Abstract

**Background:**

Oral microbiome played an important role in maintaining healthy state and might exhibit certain changes under circumstances of diseases. However, current microbiological research using sequencing techniques did not regard dead bacteria as a separate part, causing findings based on subsequent analyses on dynamic equilibrium and functional pathways of microbes somewhat questionable. Since treatment by propidium monoazide (PMA) was able to remove dead bacteria effectively, it would be worth studying how the sequencing results after PMA treatment differed from those focusing on the whole microbiota.

**Methods:**

Unstimulated whole saliva samples were obtained from 18 healthy people from 3 age groups (children, adults, and the elderly). After removal of dead bacteria by propidium monoazide (PMA), changes in the profile of salivary microbiome were detected using *16S rRNA sequencing* technology, and differences among age groups were compared subsequently.

**Results:**

Dead bacteria accounted for nearly a half of the whole bacteria flora in saliva, while freezing had little effect on the proportion of deaths. After treatment with PMA, the numbers of OTUs reduced by 4.4–14.2%, while the Shannon diversity indices decreased significantly (*P* < 0.01). Only 35.2% of positive and 6.1% of negative correlations were found to be shared by the whole microbiota and that with dead bacteria removed. Differences in significantly changed OTUs and functional pathways among different age groups were also observed between the group of PMA and the control.

**Conclusions:**

It was necessary to take the influence of living state of bacteria into account in analytic studies of salivary microbiome.

**Supplementary Information:**

The online version contains supplementary material available at 10.1186/s12903-021-01832-5.

## Background

Oral microbiome was highly complex, constituting the second most diverse microbiota throughout the human body [1]. These bacteria had closely associated with a series of oral and systemic diseases [2]. However, there were a lot of dead bacteria in the oral cavity, including those introduced by food intake and those existed in dental plaque. Normally, diseases could only be caused by living microbes that were able to replicate [3]. Live bacteria also play important biological processes such as digestion, absorption [4] and immune responses [5, 6]. This implied that the living state of bacteria might be an important factor of various physiological and pathological processes in the oral cavity.

In the past decades, great achievements had been made in research of human microbiome using DNA sequencing method, particularly for its role in disease conditions. Nevertheless, when we performed high-throughput sequencing analyses on oral samples, the dead microbes were not separated and would be also involved in the final results. This could cause inaccuracy in our understanding of the actual composition and functional pathways of microbial communities. In other words, DNA-based identification methods could not distinguish the DNA of living microbial cells (dormant cells, and growing or non-growing metabolically active cells) from dead ones. In consideration of the slow decay rate of DNA in dead bacterial cells, DNA-based detection methods tended to overestimate the richness and abundance of bacteria in samples. Therefore, the false-positive results of living pathogens made it doubtful for exploring relationship between the disease state and oral microbiota.

In this case, propidium monoazide (PMA) was introduced on account of its highly selective ability that could penetrate into the membranes of the damaged cells and covalently combined with the DNA in form of cross-linking [7]. PMA could effectively inhibit the amplification of DNA from the dead cells of both Gram-negative and Gram-positive bacteria [8–10] during the polymerase chain reaction (PCR) process prior to the following community sequencing procedures. PMA had been used in microbiological studies with regard to low-biomass cleanroom conditions [11], respiratory tract [12], and improvement of the counting accuracy of samples treated with sterilants [13, 14].

Saliva, as a pool of microorganisms with advantages of non-invasive and simple sampling procedures, had become a hotspot and representative of oral microbiome research [15, 16]. One recent study [17] reported that PMA treatment had “subtle” effects on taxonomic composition of unstimulated saliva, which would be much less significant than the influences of temporal dynamics or individual specificity. Hence, the present study was designed to use *16S rRNA* sequencing technique to make comparisons between the whole bacteria flora in saliva and that with dead bacteria removed with PMA employed as the separating agent. The aspects of comparisons included not only the composition of salivary microbiome but also the correlations and functional pathways, as well as potential age-effects. In one word, the main purpose of our study was to investigate if removal of dead bacteria by PMA treatment had effects on the profile of salivary microbiome.

## Methods

### Sample information

Unstimulated whole saliva samples were collected from participants aged 4–6, 18–30, and 50–60 years, with the exclusion criteria as follows: (1) who had taken antibiotics or probiotics within the last 4 weeks before sampling; (2) who were undergoing orthodontic treatment; (3) who could not cooperate with the study process. This study was approved by the ethics committee of Peking University School of Stomatology (PKUSSIRB-202056084). All participants had signed informed consent by themselves or their guardians before sampling procedures started.

### Exploration of the influence of freezing by staining of dead bacteria

We first designed a pilot study to see if freezing had impact on the ratio of dead microbes. The sampling procedures began in the morning at 8:00–10:00 a.m., and no food, water, chewing gum or oral cleaning measures were taken within 2 h before sampling. After rinsing mouth with pure water thoroughly and having a rest for 10 min, 2 ml unstimulated whole saliva was collected from each of the 3 healthy participants aged 18–30 years. These saliva samples were collected by 2 separate tubes (1 ml saliva in each tube) and labelled with different group names (Group Im and Group Fr, for grouping details please see below). All the samples were centrifuged at 5000 rpm × 10 min, then the supernatant was discarded. Samples in Group Im were immediately stained with the LIVE/DEAD™ BacLight™ Bacterial viability and counting Kit (Thermo Fisher Scientific, USA), whereas samples in Group Fr were kept in the refrigerator at − 80 °C for 5 days before the staining procedures began. According to the instructions, 2 ml Component A (SYTO 9 dye) and 2 ml Component B (Propidium iodide) were mixed in a microfuge tube thoroughly first, then the dye mixture was added to ddH_2_O with the concentration of 3 μl/ml. Afterwards, 300 μl of the prepared mixture was added to resuspend the precipitate. The solution was mixed thoroughly and incubate at room temperature in a dark environment for 15 min. 20 μl stained solution was placed on the slide, fixed by the cover slide, and observed under the fluorescence confocal microscope (Leica, TCS SP8, Germany; × 63 with an additional × 5 zoom) which excitation/emission wavelengths were set at 488/500–550 nm for SYTO 9 and 561/575–650 nm for propidium iodide. CLSM images were acquired by the software LAS X at a resolution of 512 × 512 pixels. Six 5 × zoom fields of view were randomly selected, and the dead bacteria ratios were calculated by Image J 1.53 and compared between Group Im and Group Fr to see if freezing did affect the proportions of deaths.

### Sampling and preparation of saliva

18 participants covering age groups of 4–6, 18–30, 50–60 years old were recruited (3 males and 3 females in each age group; Group C1 and P1: 4–6-year-olds as a representative of children; Group C2 and P2: 18–30-year-olds on behalf of adults; Group C3 and P3: 50–60-year-olds representing the elderly; see Additional file 1: Table S1 for more information). “Group C” represented the mixture of Group C1, C2 and C3, while “Group P” was the summary of Group P1, P2 and P3.

Unstimulated whole saliva was selected as the sample for the present study to avoid any potential effects brought by extraneous stimulating method. The sampling procedures also began in the morning at 8:00–10:00 a.m., and no food, water, chewing gum or oral cleaning measures were taken within 2 h before sampling. After rinsing mouth with pure water thoroughly and having a rest for 10 min, 2 ml unstimulated whole saliva was collected from each participant. These saliva samples were collected by 2 separate tubes (1 ml saliva in each tube), labelled with different group names (Group C for control, and Group P for PMA treatment) and placed on ice at once.

### PMA treatment and DNA extraction

In Group P, saliva samples were centrifuged at 7000 rpm for 5 min, then the supernatant was discarded and the precipitate were rinsed with 1 ml PBS for once. A small amount of the sample was extracted and counted under microscope, then the sample was diluted with 1 × PBS to ensure that the concentration of bacteria was around 1 × 10^7^–2 × 10^7^/ml. 1.25 μl PMA solution (20 mM) was added to 500 μl diluted saliva sample, then the mixture was incubated for 5 min without light under intermittent oscillation and placed under a 650 W halogen lamp for 3 min with a distance of 6 cm away from the light source. The PMA-treated samples were centrifuged at 7000 rpm for 5 min and the supernatant was removed again, and the precipitate was rinsed with 1 ml PBS for once before genomic DNA extraction in terms of the research protocol by Sweet et al. [18].

### PCR amplification and sequencing

The sequence of the V3-V4 region of *16S rRNA* was analysed by high-throughput sequencing. A two-step PCR amplification method was used to construct the library. The purified DNA was used as a template to be amplified by *16S rRNA* V3-V4 region universal primer 357F (5′-ACTCCTACGGRAGGCAGCAG-3′) and 806R (5′-GGACTACHVGGGTWTCTAAT-3′) and detected via 1.2% agarose gel electrophoresis. The samples with good detection efficiency were recycled from the agarose gel electrophoresis, and 8 cycles of PCR amplification were carried out with the recovered product as a template. The sequences needed for Illumina platform sequencing was added to both ends of the target fragment. All the PCR products were recycled by AxyPrepDNA gel Recovery Kit (AXYGEN company) and quantified by FTC-3000TM Real-Time PCR. After the mole ratio was mixed, the library was constructed and sequenced on the Illumina platform.

The initial PCR mixtures contained 5× Buffer 10 μl, dNTP (10 mM) 1 μl, Phusion super fidelity DNA polymerase 1U, forward and reverse primers (10 mM) 1 μl, template DNA 20-50 ng, supplemental ultra-pure water to 50 μl. Thermal cycling consisted of an initial denaturation step at 94 °C for 2 min, followed by 24 cycles of denaturation 94 °C for 30 s, annealing at 56 °C for 30 s, and extension at 72 °C for 30 s, with a final extension step at 72 °C for 5 min. The secondary PCR mixtures contained 5× Buffer 8 μl, dNTP (10 mM) 1 μl, Phusion super fidelity DNA polymerase 0.8U, positive and reverse primers (10 mM) 1 μl, template DNA 5 μl, supplement ultra-pure water to 40 μl. Thermal cycling had similar procedures with the above, but the number of cycles were 8 and a heat preservation step at 10 °C was supplemented in the end.

### Analysis of operational taxonomic units (OTUs) profile

The raw sequences were processed to concatenate reads into tags according to their overlaps, after which reads belonging to each sample were separated with barcodes and low-quality reads were removed. The processed tags were clustered, and chimaeras were removed prior to analysis. Operational taxonomic units (OTUs) were defined at 97% sequence similarity to taxa by matching to the Silva128 database using mothur (classify.seqs) software with a confidence threshold of 0.6.

### Statistical analysis

The Venn diagrams were drawn by jvenn method [19]. For alpha diversity analysis, nonparametric tests (Wilcox rank-sum test for two groups and Kruskal–Wallis test for multiple groups) were used, and the results were drawn by the boxplot package of R software (version: 3.6.1). For beta diversity analysis, UniFrac method was used to obtain the distance matrix between samples, and the multi-sample similarity tree was drawn using the dendextend package of R. Also, PERMANOVA (vegan-version: 2.5–5, function: adnois) was conducted in R software with gender and age group selected as covariates to compare the difference.

At the genus level, the ggplot2 package of R was employed to plot the column chart and draw the column diagram of microbiota. Genus count table was used as an input for SparCC. The SparCC analysis was conducted with composition-robust correlations from the median of 20 iterations, while 1000 bootstrap samples were used to infer pseudo *P* values with SpiecEasi (version 1.1.1) of R. The inferred correlations were restricted to those with a correlation co-efficient of more than 0.6 or less than −0.6 (*P* < 0.05, two-sided). Visualization of results was performed with igraph (version 1.2.5) package of R. The Picrust2 (phylogenetic investigation of communities by reconstruction of unobserved states) was used to identify differentially abundant OTUs and functional pathways.

The aldex2 (anova-like differential expression 2, version 1.18.0) package of R was carried out to identify differentially abundant OUTs and functional pathways, with nonparametric tests used in comparisons between different groups. OTUs and functional pathways with a *P* value of less than 0.05 were screened, and GGplot2 (version 3.3.2) package of R was used for visualization of the results.

## Results

### Influence of freezing on the dead ratio of salivary microbiota

**Fig. 1:**
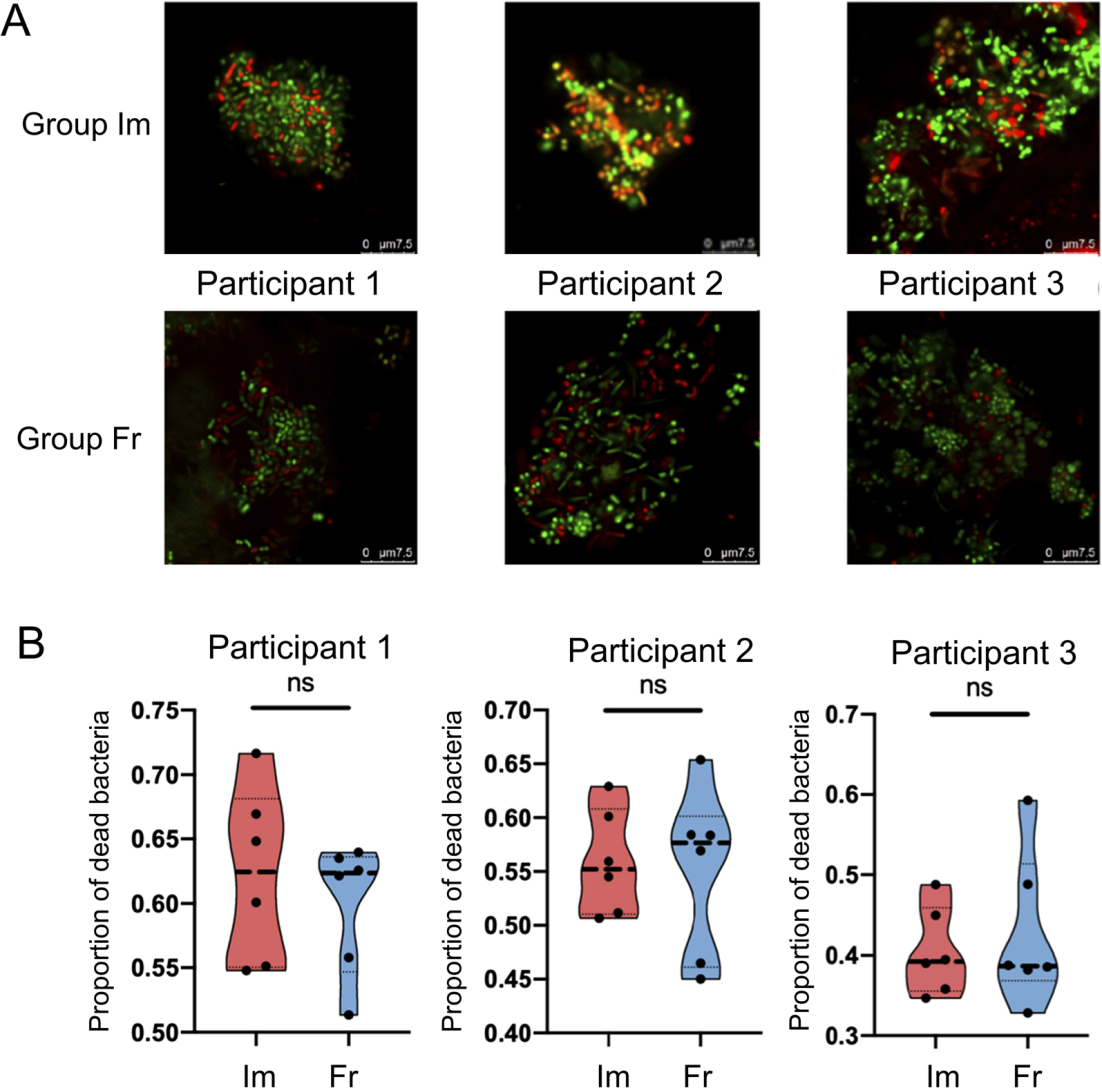
The living state and dead proportion of salivary microbiome. **A** Fluorescence microscopic observation with staining immediately (upper row, Group Im) or after keeping in freezing condition (−80 °C) for 5 days (lower row, Group Fr). **B** The proportion of dead bacteria in the three participants (*P* values: 0.51, 0.84, 0.62). Error bars represented median ± quartile (n = 6)

Figure 1A showed representative fluorescence images of the living state of salivary microbiome, with a dead proportion of 32.8–71.7% (Fig. 1B). No significant difference was found between immediately stained samples and those undergone freezing condition.

### Changes in genera profile after PMA treatment

**Fig. 2:**
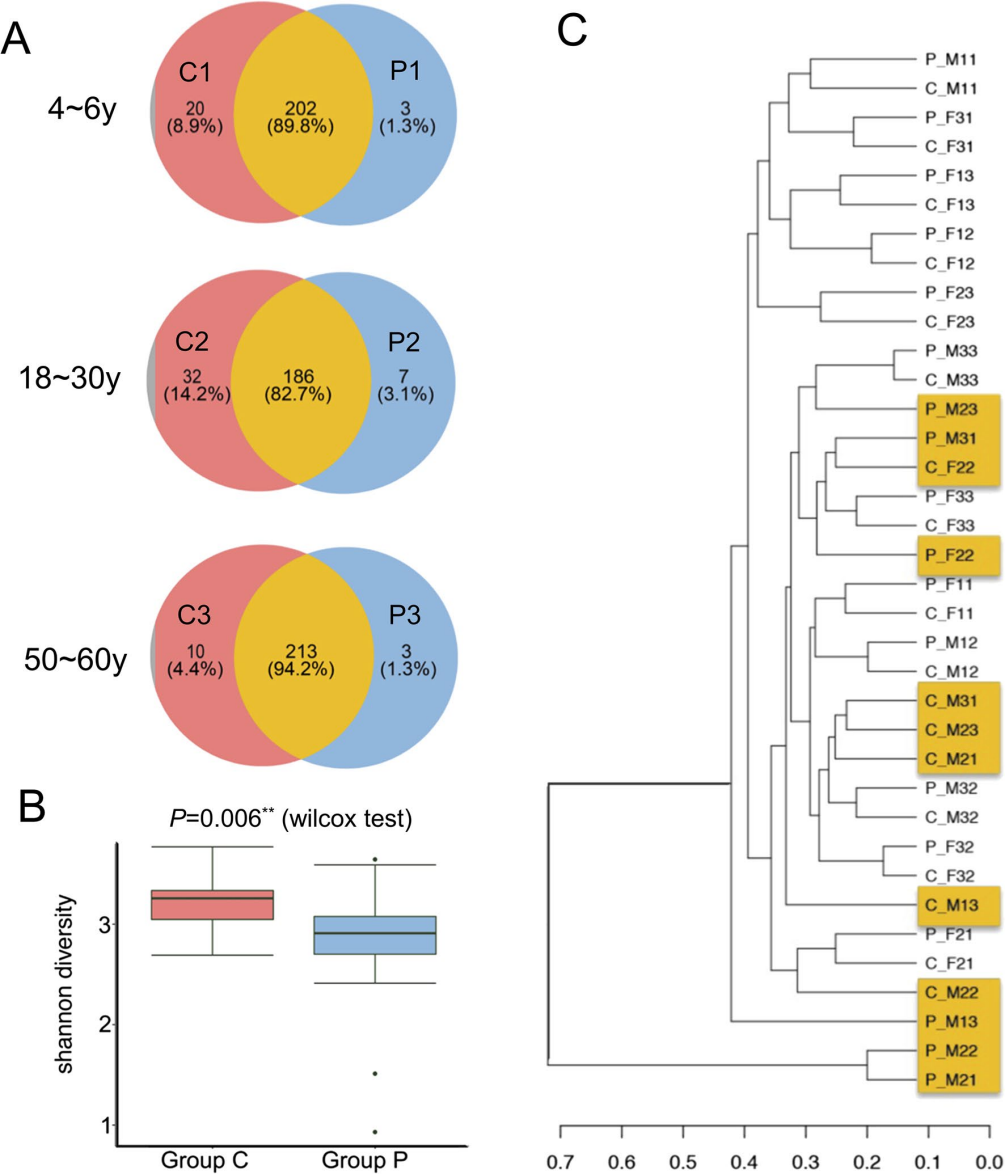
The genera profiles of salivary microbiome in pre- and post-PMA-treatment groups. **A** The Venn diagrams of Group C (control) and P (with PMA treatment) in the three age groups. **B** Comparison of α-diversity in terms of the Shannon indices in Group C and P. **P ≤ 0.01. **C** Dendrogram of all the 18 samples

Total number of raw reads per sample was provided in Additional file 1: Table S2, and the mean reads per sample after QC was 32301. The sequencing depth was analysed and a sparse curve was drawn (Additional file 1: Figure S1). After treated with PMA, the numbers of OTUs reduced by 4.4–14.2% (Fig. 2A and Additional file 1: Table S3). The Shannon index for α-diversity decreased significantly (*P* = 0.006, Fig. 2B) in all the three age groups. Two OTUs (Treponema sp.HMT_927 and Clostridiales_[F-1][G-1]bacterium_HMT_093) completely disappeared in Group P.

**Fig. 3:**
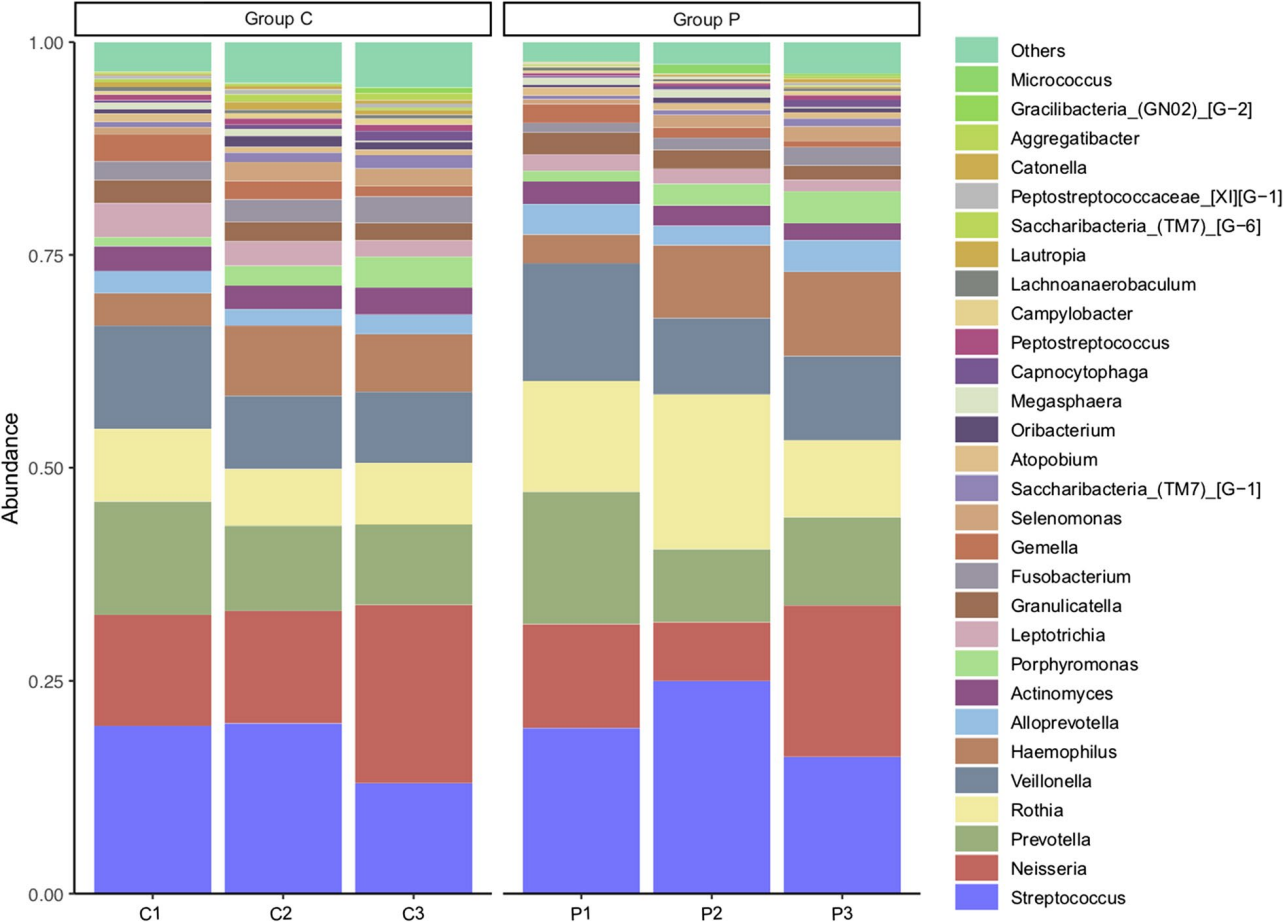
Relative abundance of genera in different groups. Group C1 & P1, participants aged 4–6 years; Group C2 & P2, participants aged 18–30 years; Group C3 & P3, participants aged 50–60 years

Comparative analysis based on the composition of genera in samples with and without PMA treatment showed that some samples derived from the same participant were separated with a distance in the cluster tree, indicating that the removal of dead bacteria had influence on the profile of salivary microbiome to a certain extent (Fig. 2C). The proportion of some high-abundance genera (e.g. *Streptococcus*, *Neisseria*, *Veroniella*, *Rosella*, *Haemophilus*, and *Actinomycetes*) increased at the genus level while that of some other relatively low-abundance bacteria decreased (Fig. 3).

The unweighted UniFrac-based PERMANOVA results showed that beta diversity was not significantly correlated with gender (*P* = 0.112), age subgroup (*P* = 0.589) and PMA treatment (*P* = 0.435), but significantly correlated with age (*P* = 0.002) among individuals (Additional file 1: Table S4).

### Different patterns in co-occurrence networks with PMA treatment

**Fig. 4:**
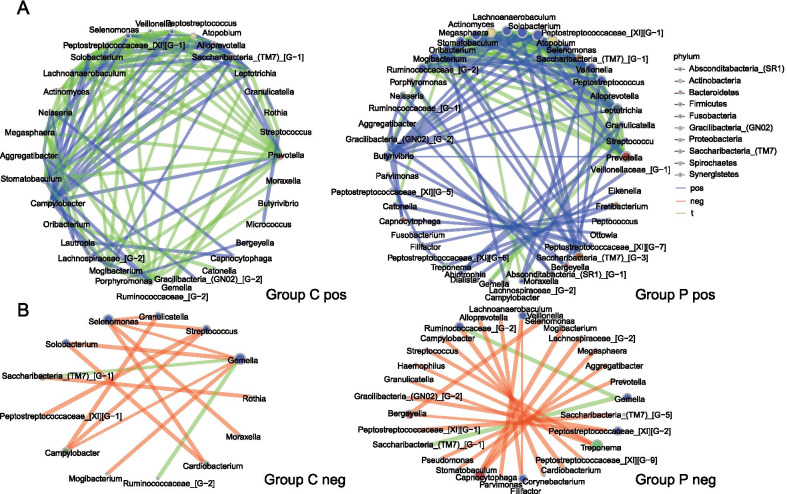
Co-occurrence networks of Group C and P. Nodes represented genera (coloured by different phylum), which size indicated the number of genera connected. Lines between nodes represented positive correlations (**A**) or negative correlations (**B**), among which those shared by both Group C and P were coloured as green, while those specific in either group were coloured as blue (positive) and red (negative), respectively

Co-occurrence network analysis was carried out to investigate the variations in relationships between different genera after using PMA (Fig. 4), which results revealed that there were dramatic changes in their correlations. Only 63 pairs in 179 positive correlations and 2 in 33 negative correlations were shared by pre- and post-PMA-treatment groups (i.e., Group C and P) under the circumstance of absolute value of correlation coefficient ≥ 0.6 and *P* value < 0.05. A larger number of positive correlations among some genera (*Butyrivibrio, Saccharibacteria_(TM7)_[G-3]*, *Leptotrichia*, *Peptostreptococcus*, *Veillonella*, *Selenomonas*, *Atopobium*, *Peptostreptococcaceae_[XI][G-1]*, *Lachnoanaerobaculum*, *Actinomyces*, and *Mogibacterium*) and fewer negative correlations (mainly related to *Treponema* and *Capnocytophaga*) were found in Group P compared with Group C.

### Differences among the three age groups

**Fig. 5:**
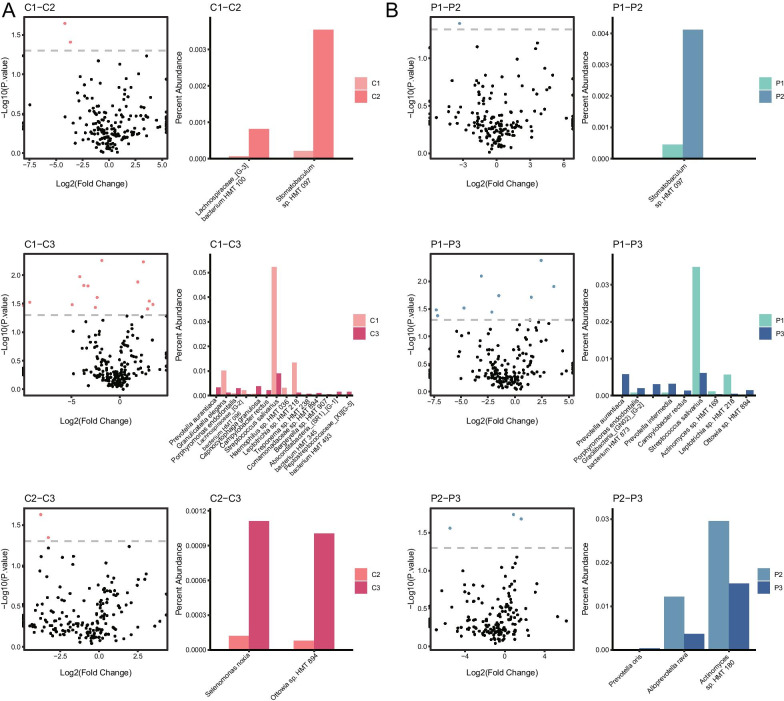
Differences of OTUs among the three age groups. Scatter diagrams and histograms showed differences at the OUT level between every two age groups of Group C (**A**) and P (**B**). Red dots in the scatter diagrams indicated that the relative abundance of these OTUs were significantly different between the two groups, while the percent abundance of these OTUs were showed in the histograms

Potential age-effects were explored by comparison among the three age groups in Group P and C. At the OTU level, some OTUs had significant differences in relative abundance between Group C1 and C2 (*Stomatobaculum sp.HMT 097*, *Lachnospiraceae_[G-3] bacterium HMT 100*), Group C1 and C3 (*Peptostreptococcaceae_[XI][G-5] bacterium HMT 493*, *Absconditabacteria_(SR1)_[G-1] bacterium HMT 345*, *Bergeyella sp. HMT 907*, *Comamonadaceae sp. HMT 894*, *Treponema sp. HMT 238*, *Leptotrichia sp. HMT 218*, *Haemophilus sp. HMT 036*, *Streptococcus salivarius*, *Campylobacter rectus*, *Capnocytophaga granulosa*, *Lachnospiraceae_[G-2] bacterium HMT 096*, *Porphyromonas endodontalis*, *Granulicatella elegans*, *Prevotalla aurantiaca*), and Group C2 and C3 (*Ottowia sp. HMT 894*, *Selenomonas noxia*). After PMA treatment, OTUs which had significant differences in relative abundance became partly different in comparisons between Group P1 and P2 (*Stomatobaculum sp.HMT 097*), Group P1 and P3 (*Ottowia sp. HMT 894*, *Leptotrichia sp. HMT 218*, *Actinomyces sp. HMT 169*, *Streptococcus salivarius*, *Campylobacter rectus*, *Prevotella intermedia*, *Gracilibacteria_(GN02) [G-2] bacterium HMT 873*, *Porphyromonas endodontalis*, *Prevotella aurantiaca*), and Group P2 and P3 (*Actinomyces sp. HMT 180*, *Alloprevotella rava*, *Prevotella oris*) (Fig. 5).

**Fig. 6:**
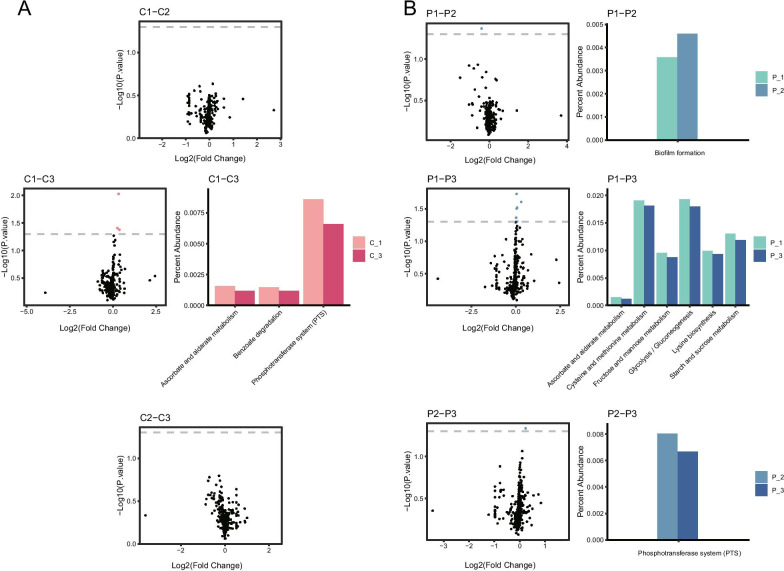
Differences of functional pathways among the three age groups. Scatter diagrams and histograms showed differences at the pathway level between every two age groups of Group C (**A**) and P (**B**). Red dots in the scatter diagrams, same as Fig. 5. The percent abundance of these pathways were also showed in the histograms

As for functional pathways, no significant difference was present in the comparison between Group C1 and C2, as well as that between Group C2 and C3, while three pathways (Phosphotransferase system-ko02060, Benzoate degradation-ko00362, Ascorbate and aldarate metabolism-ko00053) exhibited significant differences between Group C1 and C3. After PMA treatment, only one pathway was found to have significant differences in the comparison between Group P1 and P2 (Biofilm formation Escherichia coli-ko02026), so as that between Group P2 and P3 (Phosphotransferase system-ko02060). Six pathways (Starch and sucrose metabolism-ko00500, Lysine biosynthesis-ko00300, Glycolysis / Gluconeogenesis-ko00010, Fructose and mannose metabolism-ko00051, Cysteine and methionine metabolism-ko00270, Ascorbate and aldarate metabolism-ko00053) had significant differences in the comparison between Group P1 and P3 (Fig. 6).

## Discussion

The *16S rRNA* approach was a popular technique for microbiome research, however less attention was paid to the living state of bacteria which might potentially affect the results. PMA was able to remove dead microbes by covalently binding with DNA, while the viable cells, including viable-but-non-culturable (VBNC) cells which had intact cell membranes, would not be significantly affected under certain PMA concentration ranges [20]. As PMA was already used in some *in-vitro* studies and exhibited acceptable anti-bacteria effectiveness [21, 22], while one recent study reported that PMA treatment had “subtle” effects on taxonomic composition of unstimulated saliva and would be much less significant than the influences of temporal dynamics or individual specificity [17], we designed the present study to justify if removal of dead bacteria with PMA treatment could give rise to changes not only in the composition of salivary microbiome but also in the correlations and functional pathways, as well as presence of potential age-effects. Since the saliva samples might have a frozen-thawed procedure on various occasions prior to further examinations, we compared the dead ratios of salivary microbiota between fresh saliva sample and that with freezing storage via a pilot study with staining methods, which verified that freezing condition contributed little to the proportion of dead bacteria.

To our knowledge, many bacteria could not be cultured in vitro*,* resulting in a failure of simulating the actual status of living microbiota by cultivation only [23]. In the meantime, the rRNA analysis, though was used as a general indicator of active microorganisms in environmental samples, could be ineffective in certain cases [24]. It was confirmed by several previous studies that PMA was effective in sterilization of samples, during which process dead bacteria were almost completely removed with only less than 0.27% left [25–27] and without evident loss and decomposition of living bacteria [28], making PMA treatment reasonable and acceptable for separating bacteria based on the living state to keep living bacteria only in the following analysing procedures.

Besides PMA, Ethidium monoazide (EMA) [29], Surface-Enhanced Raman Scattering (SERS) [30], hand-held double monochromator [31] and RNA-based *16S rRNA* sequencing [32] could also be used to separate or remove dead bacteria. Among them, high concentration of EMA might enter the whole cells of some species, causing the lack of specificity for intact bacterial cells [7]. SERS could quantify the percentage of dead bacteria, but could not distinguish different genera [30]. Also, hand-held double monochromator had limited capacity in distinguishing different genera in mixed oral samples. Although the detection of RNA (especially the highly unstable mRNA) tended to indicate the presence of living cells far better than the detection of DNA, the degradation of RNA and the complicated physiological status of the cells both made it challenging to be used in oral samples like saliva. In addition, false-positive signals from residual transcripts could occur in case of high level of dead bacteria [7]. Among all these methods, PMA treatment was easier to operate and had reliable effects in removal of dead bacteria, which should be appropriate to be chosen as the key agent for the present study.

Our results of changes in genera profile revealed that there were a considerable proportion of dead bacteria in the whole microbiota. However, different trends were observed in the changes of genera after PMA treatment. The proportion of some high-abundance genera increased, while that of relatively low-abundance bacteria decreased. These findings implied that the role of low-abundance genera might be exaggerated in sequencing analysis without removal of dead bacteria. Besides, we observed dramatic changes in co-occurrence networks between pre- and post-PMA-treatment groups, with a greater number of positive correlations among certain genera detected in the group of PMA. *Selenomonas* was found to be significantly associated with recurrent aphthous stomatitis (RAS) [33], gestational diabetes mellitus [34], and the presence of dental caries [35] and periodontitis [36]. *Butyrivibrio* exhibited significantly higher prevalence in fecal samples collected from patients with Parkinson's Disease [37]. *Peptostreptococcus* was significantly enriched in pathological samples from patients with oral squamous cell carcinoma or periodontitis [38]. For analyses of salivary microbiome without removing dead microbes, the functions of these genera above were much likely to be underestimated. *Capnocytophaga*, which was formerly considered as a part of normal flora in the oral cavity, still had broad negative correlations after PMA treatment, demonstrating its functional potentiality in maintaining the stability of microbiota in saliva.

Among the three age groups, differences in significantly changed OTUs and functional pathways in response to PMA treatment were also observed. For these OTUs, previous studies showed that *Selenomonas noxia* in saliva was associated with refractory periodontitis [39] and hepatitis C [40], while *Capnocytophaga granulosa* was associated with Papillon-Lefèvre syndrome [41], but the associations of *Peptostreptococcaceae_[XI][G-5]_bacterium_HMT_493*, *Ottowia sp._HMT_894*, *Treponema sp._HMT_238*, *Lachnospiraceae_[G-2] bacterium HMT 096* and some other bacteria with diseases were still not clear. Some of these microbes might always come up in a dead form in saliva samples, resulting in a potential bias in analysis of the pathogenic process in the circumstances of diseases. Similar with a previous study which found certain pathways could change with age [42], we also found that some functional pathways exhibited an age difference. For example, the phosphotransferase system, which was a major mechanism used by bacteria for uptake of carbohydrates (particularly hexoses, hexitols, and disaccharides) [43], had significant differences between children (Group C1) and older people (Group C3) only before PMA treatment. Future studies with a larger number of samples would be beneficial to conduct more in-depth investigations on these age-varied pathways.

Although we confirmed the influence of removing dead bacteria by PMA on the profile of salivary microbiome in the present study, certain limitations should be kept in mind to carry forward future studies. First, saliva was selected as a representative habitat of oral microbiome in the present study, but other sites, such as dental plaque which also contained a large amount of dead bacteria [44], yet need further exploration. Second, whether removal of dead bacteria had influence on the profile of microbiome in other sequencing methods (e.g. metagenomic analysis) still remained unrevealed. Also, further studies with RNA-based 16S amplicon library included would be a much important route to validate the effects of propidium monoazide treatment. Third, since our study was based on healthy individuals, the change of salivary microbiota and the impact of PMA under circumstances of diseases (including oral and systemic diseases) could also become a future direction of research in this field. Fourth, the sample size should be enlarged with qPCR quantification applied in future studies, so as to understand the specific reduction of each strain and to learn more about whether salivary microbiota of certain population group (e.g. 18–30-year-old men, who might have much lower sequence readouts as shown in Additional file 1: Figure S1) would have some particular characteristics and hence exhibit greater changes after PMA treatment.

## Conclusions

In summary, differences in genus, diversity, correlations and functional pathways and distinctions among different age groups were found between the whole salivary microbiota and that with dead bacteria removed by PMA treatment, highlighting the potential significance of removing dead bacteria in high-throughput sequencing research. These findings enlightened us that it was necessary to take the influence of living state of bacteria into account in analytic studies of salivary microbiome.

## Supplementary Information

**Additional file 1**. **Table S1.** Information of participants and samples. **Table S2.** Total number of reads per sample. **Table S3.** Comparison among the three age groups by OTUs present in Group C and absent in Group P. **Table S4.** Beta diversity analysis based on PERMANOVA. **Figure S1.** Rarefaction curves for each experimental sample.

## Data Availability

The sequencing data from this study have been submitted to Sequence Read Archive (http://www.ncbi.nlm.nih.gov/sra/) under accession numbers PRJNA639033.

## Abbreviations

EMA: Ethidium monoazide
OTUs: Operational taxonomic units
PCR: Polymerase chain reaction
PMA: Propidium monoazide
RAS: Recurrent aphthous stomatitis
SERS: Surface-Enhanced Raman Scattering
