# Supplementary material for: The effects of removing dead bacteria by propidium monoazide on the profile of salivary microbiome
Source: BMC Oral Health. 2021 Sep 22;21:460. doi: 10.1186/s12903-021-01832-5 (PMC8456568; doi:10.1186/s12903-021-01832-5)
Supplement: Supplementary file 1 — Additional file 1. Table S1. Information of participants and samples. Table S2. Total number of reads per sample. Table S3. Comparison among the three age groups by OTUs present in Group C and absent in Group P. Table S4. Beta diversity analysis based on PERMANOVA. Figure S1. Rarefaction curves for each experimental sample. [file 12903_2021_1832_MOESM1_ESM.docx]

Supplementary document for

**The effects of removing dead bacteria by propidium monoazide on** **the profile of salivary microbiome**

Qidi Ren^#, a^, Fangqiao Wei^#, a^, Chao Yuan^a^, Ce Zhu^a, b^, Qian Zhang^c^, Junkang Quan^a^, Xiangyu Sun^*, a^, Shuguo Zheng^*, a^

**Table S1. Information of participants and samples**

| **Sample ID** | **Sex** | **Age** | **Subgroup** |
| --- | --- | --- | --- |
| C_M11 | Male | 6 | C1 (4~6y) |
| C_M12 | Male | 6 | C1 (4~6y) |
| C_M13 | Male | 6 | C1 (4~6y) |
| C_F11 | Female | 6 | C1 (4~6y) |
| C_F12 | Female | 6 | C1 (4~6y) |
| C_F13 | Female | 6 | C1 (4~6y) |
| C_M21 | Male | 28 | C2 (18~30y) |
| C_M22 | Male | 24 | C2 (18~30y) |
| C_M23 | Male | 29 | C2 (18~30y) |
| C_F21 | Female | 23 | C2 (18~30y) |
| C_F22 | Female | 29 | C2 (18~30y) |
| C_F23 | Female | 26 | C2 (18~30y) |
| C_M31 | Male | 50 | C3 (50~60y) |
| C_M32 | Male | 50 | C3 (50~60y) |
| C_M33 | Male | 51 | C3 (50~60y) |
| C_F31 | Female | 50 | C3 (50~60y) |
| C_F32 | Female | 50 | C3 (50~60y) |
| C_F33 | Female | 50 | C3 (50~60y) |
| P_M11 | Male | 6 | P1 (4~6y) |
| P_M12 | Male | 6 | P1 (4~6y) |
| P_M13 | Male | 6 | P1 (4~6y) |
| P_F11 | Female | 6 | P1 (4~6y) |
| P_F12 | Female | 6 | P1 (4~6y) |
| P_F13 | Female | 6 | P1 (4~6y) |
| P_M21 | Male | 28 | P2 (18~30y) |
| P_M22 | Male | 24 | P2 (18~30y) |
| P_M23 | Male | 29 | P2 (18~30y) |
| P_F21 | Female | 23 | P2 (18~30y) |
| P_F22 | Female | 29 | P2 (18~30y) |
| P_F23 | Female | 26 | P2 (18~30y) |
| P_M31 | Male | 50 | P3 (50~60y) |
| P_M32 | Male | 50 | P3 (50~60y) |
| P_M33 | Male | 51 | P3 (50~60y) |
| P_F31 | Female | 50 | P3 (50~60y) |
| P_F32 | Female | 50 | P3 (50~60y) |
| P_F33 | Female | 50 | P3 (50~60y) |

**Table S2. Total number of reads per sample**

| sample ID | raw reads | after QC |
| --- | --- | --- |
| C_M11 | 36937 | 33451 |
| C_M12 | 37692 | 34470 |
| C_M13 | 32574 | 30363 |
| C_F11 | 33057 | 30098 |
| C_F12 | 30971 | 27883 |
| C_F13 | 39055 | 36309 |
| C_M21 | 41981 | 38815 |
| C_M22 | 30338 | 28110 |
| C_M23 | 34338 | 30647 |
| C_F21 | 33459 | 30697 |
| C_F22 | 35664 | 33098 |
| C_F23 | 37234 | 33078 |
| C_M31 | 44875 | 40860 |
| C_M32 | 32964 | 29862 |
| C_M33 | 37396 | 34397 |
| C_F31 | 35195 | 31888 |
| C_F32 | 35928 | 32824 |
| C_F33 | 39125 | 36404 |
| P_M11 | 35640 | 33197 |
| P_M12 | 31889 | 29281 |
| P_M13 | 32143 | 30187 |
| P_F11 | 35724 | 32653 |
| P_F12 | 42793 | 39202 |
| P_F13 | 36622 | 34192 |
| P_M21 | 34484 | 32306 |
| P_M22 | 32185 | 30611 |
| P_M23 | 31427 | 29451 |
| P_F21 | 35407 | 32563 |
| P_F22 | 30520 | 28428 |
| P_F23 | 40549 | 37329 |
| P_M31 | 33792 | 31402 |
| P_M32 | 38250 | 35247 |
| P_M33 | 33480 | 30999 |
| P_F31 | 34390 | 31828 |
| P_F32 | 29044 | 26990 |
| P_F33 | 25123 | 23721 |

**Table S3. Comparison among the three age groups by OTUs present in Group C and absent in Group P**

(A) Summary of specific OTUs in for each age group

| **OTU number** | **Name** | **Level** |
| --- | --- | --- |
| 4~6y | | |
| OTU247 | pittmaniae | species |
| OTU235 | diminuta | species |
| OTU195 | gingivicola | species |
| OTU182 | artemidis | species |
| OTU173 | sp._HMT_315 | species |
| OTU171 | sp._HMT_308 | species |
| OTU175 | Bergeyella | genus |
| 18~30y | | |
| OTU252 | sp._HMT_894 | species |
| OTU237 | sakazakii | species |
| OTU234 | sp._HMT_266 | species |
| OTU226 | curtum | species |
| OTU223 | ignava | species |
| OTU213 | vestrisii | species |
| OTU208 | sp._HMT_927 | species |
| OTU187 | sp._HMT_183 | species |
| OTU183 | sp._HMT_080 | species |
| OTU180 | dentalis | species |
| OTU176 | Treponema | species |
| OTU172 | massiliensis | species |
| OTU170 | [G-1]_bacterium_HMT_155 | species |
| OTU167 | pleuritidis | species |
| OTU160 | [G-5]_bacterium_HMT_505 | species |
| OTU137 | [XI][G-4]_bacterium_HMT_369 | species |
| OTU134 | coli | species |
| OTU123 | (TM7)_[G-1]_bacterium_HMT_346 | species |
| OTU102 | [XI][G-6]_nodatum | species |
| OTU86 | Fusobacterium | genus |
| OTU185 | Corynebacterium | genus |
| OTU169 | Selenomonas | genus |
| OTU101 | Sneathia | genus |
| 50~60y | | |
| OTU109 | capitis | species |
| OTU236 | sp._HMT_166 | species |
| OTU242 | [G-2]_bacterium_HMT_088 | species |
| OTU255 | omnicolens | species |
| OTU202 | Coriobacteriaceae | family |

(B) Summary of OTUs shared by ≥ 2 age groups

| **OTU number** | **Name** | **Level** |
| --- | --- | --- |
| 4~6y | | |
| OTU61 | luteus | species |
| OTU262 | (GN02)_[G-1]_bacterium_HMT_871 | species |
| OTU250 | sp._HMT_847 | species |
| OTU239 | muelleri | species |
| OTU225 | [F-1][G-1]_bacterium_HMT_093 | species |
| OTU224 | sp._HMT_262 | species |
| OTU208 | sp._HMT_927 | species |
| OTU147 | sp._HMT_257 | species |
| OTU136 | (TM7)_[G-2]_bacterium_HMT_350 | species |
| OTU251 | Lachnospiraceae_[XIV] | family |
| OTU221 | OTU221 | OTU |
| OTU193 | OTU193 | OTU |
| OTU192 | OTU192 | OTU |
| 18~30y | | |
| OTU262 | (GN02)_[G-1]_bacterium_HMT_871 | species |
| OTU250 | sp._HMT_847 | species |
| OTU239 | muelleri | species |
| OTU225 | [F-1][G-1]_bacterium_HMT_093 | species |
| OTU224 | sp._HMT_262 | species |
| OTU147 | sp._HMT_257 | species |
| OTU136 | (TM7)_[G-2]_bacterium_HMT_350 | species |
| OTU221 | OTU221 | OTU |
| OTU193 | OTU193 | OTU |
| 50~60y | | |
| OTU208 | sp._HMT_927 | species |
| OTU225 | [F-1][G-1]_bacterium_HMT_093 | species |
| OTU61 | luteus | species |
| OTU251 | Lachnospiraceae_[XIV] | family |
| OTU192 | OTU192 | OTU |

**Table S4. Beta diversity analysis based on PERMANOVA**

|  | Df | SumsOfSqs | MeanSqs | F.Model | R2 | Pr(>F) |
| --- | --- | --- | --- | --- | --- | --- |
| Sex | 1 | 0.07411 | 0.074108 | 1.732 | 0.05699 | 0.112 |
| Age | 1 | 0.16756 | 0.167558 | 3.9161 | 0.12885 | 0.002** |
| Group | 1 | 0.04111 | 0.041108 | 0.9607 | 0.03161 | 0.435 |
| Subgroup | 4 | 0.1546 | 0.03865 | 0.9033 | 0.11888 | 0.589 |
| Sex:Age | 1 | 0.03241 | 0.032413 | 0.7575 | 0.02493 | 0.648 |
| Sex:Group | 1 | 0.00647 | 0.006467 | 0.1511 | 0.00497 | 1 |
| Age:Group | 1 | 0.00515 | 0.005154 | 0.1205 | 0.00396 | 1 |
| Sex:Subgroup | 4 | 0.11874 | 0.029685 | 0.6938 | 0.09131 | 0.887 |
| Age:Subgroup | 1 | 0.01567 | 0.01567 | 0.3662 | 0.01205 | 0.964 |
| Residuals | 16 | 0.6846 | 0.042787 |  | 0.52645 |  |
| Total | 31 | 1.30041 |  |  | 1 |  |

**Figure S1**. Rarefaction curves for each experimental sample
